# Latent class analysis of 216 patients with adult-onset Still’s disease

**DOI:** 10.1186/s13075-021-02708-3

**Published:** 2022-01-03

**Authors:** Takahiro Sugiyama, Shunsuke Furuta, Masaki Hiraguri, Kei Ikeda, Yosuke Inaba, Shin-ichiro Kagami, Yasuhiko Kita, Kei Kobayashi, Yoshihisa Kobayashi, Kazuhiro Kurasawa, Daiki Nakagomi, Yasushi Nawata, Yohei Kawasaki, Yuki Shiko, Takao Sugiyama, Hiroshi Nakajima

**Affiliations:** 1 Department of Allergy and Clinical Immunology, Chiba University Hospital, 1-8-1 Inohana, Chuo-ku, Chiba-shi, Chiba, 260-8670 Japan; 2 Department of Rheumatology and Allergy, Japanese Red Cross Narita Hospital, Chiba, Japan; 3 Biostatistics Section, Clinical Research Centre, Chiba University Hospital, Chiba, Japan; 4 Department of Allergy and Clinical Immunology, Asahi General Hospital, Chiba, Japan; 5 Department of Rheumatology, Yokohama Rosai Hospital, Yokohama, Japan; 6 Third Department of Internal Medicine, University of Yamanashi, Yamanashi, Japan; 7 Department of Internal Medicine, Chiba Aoba Municipal Hospital, Chiba, Japan; 8 Department of Rheumatology, Dokkyo Medical University, Tochigi, Japan; 9 Center for Rheumatic Diseases, Chibaken Saiseikai Narashino Hospital, Chiba, Japan; 10 Department of Rheumatology, National Hospital Organization Shimoshizu Hospital, Chiba, Japan

**Keywords:** Adult-onset Still’s disease, Macrophage activation syndrome, Latent class analysis, Outcomes, Relapse

## Abstract

**Background:**

Adult-onset Still’s disease (AOSD) is a rare systemic autoinflammatory disease which encompasses patients with heterogenous presentation and a wide range of clinical courses. In this study, we aimed to identify potential subgroups of AOSD and reveal risk factors for relapse.

**Methods:**

We included a total of 216 AOSD patients who received treatment in nine hospitals between 2000 and 2019. All patients fulfilled the Yamaguchi classification criteria. We retrospectively collected information about baseline characteristics, laboratory tests, treatment, relapse, and death. We performed latent class analysis and time-to-event analysis for relapse using the Cox proportional hazard model.

**Results:**

The median age at disease onset was 51.6 years. The median follow-up period was 36.8 months. At disease onset, 22.3% of the patients had macrophage activation syndrome. The median white blood cell count was 12,600/μL, and the median serum ferritin level was 7230 ng/mL. Systemic corticosteroids were administered in all but three patients (98.6%) and the median initial dosage of prednisolone was 40mg/day. Ninety-six patients (44.4%) were treated with concomitant immunosuppressants, and 22 (10.2%) were treated with biologics. Latent class analysis revealed that AOSD patients were divided into two subgroups: the typical group (Class 1: 71.8%) and the elderly-onset group (Class 2: 28.2%). During the follow-up period, 13 of 216 patients (6.0%) died (12 infections and one senility), and 76 of 216 patients (35.1%) experienced relapses. Overall and relapse-free survival rates at 5 years were 94.9% and 57.3%, respectively, and those rates were not significantly different between Class 1 and 2 (*p*=0.30 and *p*=0.19). Time-to-event analysis suggested higher neutrophil count, lower hemoglobin, and age ≥65 years at disease onset as risk factors for death and age ≥65 years at disease onset as a risk factor for relapse.

**Conclusions:**

AOSD patients were divided into two subgroups: the typical group and the elderly-onset group. Although the survival of patients with AOSD was generally good, the patients often experienced relapses. Age ≥65 years at disease onset was the risk factor for relapse.

**Supplementary Information:**

The online version contains supplementary material available at 10.1186/s13075-021-02708-3.

## Background

Adult-onset Still’s disease (AOSD) is a rare systemic autoinflammatory disease that affects adults with a higher prevalence in women [1–3]. In 1897, George Frederic Still first reported juvenile chronic arthritis with fever and rash as Still’s disease [4], which is now considered systemic juvenile idiopathic arthritis (sJIA). Later, Adult Still’s disease (ASD) was reported by Eric Bywaters as a series of adult patients who had features similar to the children with sJIA with three main symptoms: quotidian fevers, arthritis, and evanescent rash and did not fulfill the criteria for classic rheumatoid arthritis [5]. ASD is now considered a disease concept containing both sJIA patients after becoming adults and AOSD patients.

Clinical manifestations of AOSD include fever, rash, arthralgia, sore throat, elevated liver enzymes, and hyperferritinemia [6–8]. Above all, serum ferritin levels are typically much higher than those in other autoimmune diseases, autoinflammatory diseases, infections or neoplasms, characterized by decreased glycosylated ferritin (<20%) [9, 10]. A five-fold increase of serum ferritin levels compared with the normal value is strongly suggestive of AOSD, and ferritin is also considered a useful marker of disease activity of AOSD [11, 12]. However, AOSD patients do not always present a five-fold increase of serum ferritin levels and other manifestations of AOSD are non-specific. Considering the lack of specific disease markers, patients diagnosed as AOSD could be a heterogeneous population.

Regarding the outcomes of patients with AOSD, the mortality rate has been reported to be 2.6–5.5% [13, 14]. The AOSD patients can have severe complications such as macrophage activation syndrome (MAS), disseminated intravascular coagulation (DIC), thrombotic thrombocytopenic purpura, acute respiratory distress syndrome, and diffuse alveolar hemorrhage [15–22], which can lead to mortality. In terms of relapse, some patients with AOSD experience multiple relapses, while others maintain long-term remission without relapse. In the past, three differential clinical patterns of AOSD have been described: (1) monocyclic pattern, characterized by a single systemic episode; (2) polycyclic pattern, associated with a longer clinical course, alternating with remissions; and (3) chronic pattern, related to persistently active disease with associated polyarthritis [23]. According to the previous review, 30% of AOSD patients develop a monocyclic pattern, 30% a polycyclic pattern, and 40% a chronic pattern on average [1]. However, it is still difficult to predict which clinical pattern the patient will follow, and risk factors for relapse are still unknown.

In this retrospective observational study, we addressed two clinical questions using a large cohort of AOSD patients. First, we aimed to classify the subgroups of AOSD patients, who are considered a heterogenous population. Second, we also aimed to identify the risk factors for relapses by analyzing the clinical features at disease onset.

## Patients and methods

### Patients

AOSD patients who received treatment in nine Japanese hospitals between April 2000 and March 2019 were the subjects of this retrospective study. Fourteen patients who lacked baseline data were not included. Thirteen patients whose conditions were indistinguishable from other diseases, such as viral infection or malignant lymphoma, were not included. Consequently, a total of 216 AOSD patients who fulfilled the Yamaguchi classification criteria [24] and were 16 years or older at the time of diagnosis were included in the study.

### Data collection

The presence/absence of the following clinical features at diagnosis was determined: fever, typical rash, atypical rash, arthralgia, myalgia, sore throat, lymphadenopathy, hepatomegaly, splenomegaly, abnormal liver function tests, chest pain, and abdominal pain. We also collected the results of blood examinations at diagnosis, which included white blood cell (WBC) count, neutrophil count, hemoglobin (Hb), platelet count, erythrocyte sedimentation rate (ESR), aspartate aminotransferase (AST), alanine aminotransferase (ALT), lactate dehydrogenase (LDH), C-reactive protein (CRP), and ferritin. In addition, we assessed the presence/absence of AOSD-related specific complications at diagnosis, which included MAS, DIC, renal dysfunction, pleuritis, pericarditis, myocarditis, and interstitial pneumonia. MAS was defined following diagnostic criteria proposed by the Histocyte Society in 1991 and updated in 2004 by Fardet et al. [25–28]. We also calculated the systemic score for AOSD proposed by Pouchot et al. [29]. This score assigns one point to each of 12 manifestations: fever, typical rash, pleuritis, pneumonia, pericarditis, hepatomegaly, or abnormal liver function tests, splenomegaly, lymphadenopathy, leukocytosis > 15,000mm^3^, sore throat, myalgia, and abnormal pain. Information on the use of systemic corticosteroids, converted to the equivalent prednisolone dose, immunosuppressants, biologics, endotoxin adsorption, and plasma exchange was also collected.

We also assessed the duration of time from diagnosis of AOSD to death and to the first relapse. Relapse was defined as a state in which clinical symptoms were present and treatment was reinforced after remission; remission was defined as the absence of systemic symptoms or laboratory evidence of disease activity for at least three consecutive months, regardless of therapy.

Data were retrospectively acquired from patients’ medical charts and electronic records.

Clinical courses of the patients with AOSD were classified according to the types of monocyclic, polycyclic, or chronic pattern. The classification was based on reviews of case history by the authors according to a previous report [30]. The polycyclic pattern was defined as the existence of a symptom-free period of at least 2 months between 2 relapses. The chronic pattern was defined as the presence of persistent joint symptoms.

### Statistical analysis

Comparisons between groups were performed using the Mann-Whitney *U* test for continuous and ordinal variables and the chi-square test for categorical variables. We used latent class analysis to identify subgroups based on baseline patient/disease characteristics. A multiple-group latent class model [31] with the optimal number of computer-derived subgroups (latent classes) was determined using model selection criteria, consistent Akaike information criteria and Bayesian information criteria values. After the number of classes was chosen, individuals were assigned to the class in which they had the highest posterior probability of membership. The overall survival rates and the relapse-free survival rates were calculated using the Kaplan-Meier method and the survival rates between groups were compared by the log-rank test. To assess the potential risk factors for relapse, time-to-event analysis was performed using the univariate Cox proportional hazard, and multivariate model approach was also conducted with these risk factors included as covariates. *P*<0.05 was considered statistically significant. Latent class analysis was performed using the PROC LCA (ver 1.3.2) [32], the remaining analyses were conducted using SAS 9.4 (SAS Institute, Cary, NC, USA).

## Results

### Baseline characteristics

We identified 216 consecutive patients with AOSD. Baseline characteristics of these patients are shown in Table 1. The median age at disease onset was 51.6 years and 75.9% were female. The median WBC count at onset was 12,600/μL and the median neutrophil count was 10677/μL. Serum ferritin and CRP levels at onset were highly elevated (median 7,230ng/mL and 10.8mg/dL).

**Table 1:** Baseline characteristics of the patients

	Total (*n*=216)	Relapse (*n*=76)	Non-relapse (*n*=140)
General characteristics			
Age, median years [IQR]	51.6 [33.1–68.1]	50.3 [30.2–65.9]	47.9 [31.8–69.9]
Female, *n* (%)	164 (75.9%)	68 (89.4%)	59 (65.6%)
Follow-up time, median months [IQR]	36.8 [12.1–88.2]	80.6 [40.0–135.8]	18.8 [8–46.8]
Proportion of patients with the specific symptom			
Fever, %	99.5%	100.0%	98.9%
Rash, %	90.7%	97.3%	87.1%
Abnormal liver function tests, %	81.0%	85.5%	76.9%
Arthralgia, %	79.5%	82.6%	77.8%
Sore throat, %	63.3%	56.1%	67.1%
Lymphadenopathy, %	63.5%	60.0%	65.9%
Splenomegaly, %	49.5%	47.8%	50.4%
Chest pain, %	1.9%	1.3%	2.1%
Abdominal pain, %	1.9%	1.3%	2.1%
Laboratory tests			
White blood cell count, median /μL [IQR]	12600 [9400–18100]	12600 [9190–17975]	12630 [9575–18100]
Neutrophil count, median /μL [IQR]	10677 [7555–15794]	10583 [7235–16194]	10677 [7665–14960]
Hemoglobin, median g/dL [IQR]	11.1 [10.1–12.4]	10.8 [9.8–11.7]	11.3 [10.2–12.7]
Platelet, median /μL [IQR]	258 [163–366]	260 [170–351]	256 [159–377]
Erythrocyte sedimentation rate, median mm/h [IQR]	76 [41–94]	80 [40–95]	75 [44–92]
Serum aspartate aminotransferase, median U/L [IQR]	69 [46–128]	68 [47–125]	69 [45–130]
Serum alanine aminotransferase, median U/L [IQR]	52 [30–104]	50 [25–87]	53 [31–110]
Serum lactate dehydrogenase, median U/L [IQR]	521 [314–761]	587 [357–796]	501 [303–734]
Serum C-reactive protein, median mg/dL [IQR]	10.8 [5.7–16.6]	11 [5.8–18.6]	10.8 [5.7–14.8]
Serum ferritin, median ng/mL [IQR]	7230 [2002–22065]	8536 [1745–29940]	6190 [2201–18472]
Complications			
Macrophage activation syndrome, %	22.3%	18.4%	24.5%
Disseminated intravascular coagulation, %	13.4%	17.1%	11.5%
Renal dysfunction, %	3.7%	3.9%	3.6%
Pleuritis, %	12.5%	10.7%	13.6%
Pericarditis, %	7.4%	4.0%	9.3%
Myocarditis, %	0.0%	0.0%	0.0%
Interstitial pneumonia, %	2.3%	2.7%	2.1%

*IQR* interquartile range

Regarding symptoms, most patients had fever (99.5%), rash (90.7%), abnormal liver function tests (81.0%), and arthralgia (79.5%). Other classical symptoms, such as sore throat (63.3%), lymphadenopathy (63.5%), or splenomegaly (49.5%) were also frequently present (Table 1), which was consistent with the previous reports.

The median systemic score was five points and 38 patients had systemic scores greater than seven points, which was previously reported as a risk factor for death [33]. However, the systemic score did not correlate with death or relapse in our study (*p*=0.21 and *p*=0.15, respectively).

With regard to the pre-specified complications, 22.3% of the patients had MAS and 13.4% had DIC. Renal dysfunction and interstitial pneumonitis were rare in our cohort (3.7% and 2.3%, respectively) (Table 1). Patient characteristics according to presence/absence of MAS were summarized in Supplementary Table 1.

As to the patterns of clinical course, 136 patients (62.9%) had a monocyclic pattern, while 76 patients (35.1%) presented a polycyclic pattern. In our cohort, only four patients (1.8%) presented a chronic pattern.

### Treatments

The treatment details for the new-onset disease are shown in Table 2. Systemic corticosteroids were administered in all but three patients (98.6%) and the median initial dosage of prednisolone was 40mg/day. Ninety-six patients (44.4%) were treated with concomitant immunosuppressants, and 22 (10.2%) were treated with tocilizumab. Plasma exchange was performed in five patients (2.3%). None of the patients were treated with interleukin-1 inhibitors such as canakinumab and anakinra.

**Table 2:** Treatment details

	Treatment at disease onset (*n*=216)	Treatment after first relapse (*n*=76)
Corticosteroid, *n* (%)	213 (98.6%)	76 (100%)
Initial dose of corticosteroid, median mg/day	40	40
Pulse corticosteroid therapy, *n* (%)	41 (5.3%)	6 (7.9%)
Increased corticosteroid, *n* (%)	N/A	58 (76.3%)
Immunosuppressive agents, *n* (%)	96 (44.4%)	38 (50.0%)
Cyclosporine	76 (35.2%)	23 (30.3%)
Methotrexate	14 (6.5%)	6 (7.9%)
Tacrolimus	5 (2.3%)	5 (6.6%)
Biological agents, *n* (%)	22 (10.2%)	13 (17.1%)
Tocilizumab	22 (10.2%)	11 (14.5%)
Adalimumab	0	1 (1.3%)
Etanercept	0	1 (1.3%)
Plasma exchange, *n* (%)	5 (2.3%%)	0

*N/A* not applicable

During the follow-up period, 76 patients (35.1%) experienced relapses, of which 33 (15.2%) had multiple relapses. At the time of the first relapse, 29 of the 76 patients (38.2%) were taking prednisolone with the median of 8mg/day, while the remaining 47 (61.8%) had stopped glucocorticoids prior to the relapse. Fifty-eight of the 76 patients (76.3%) increased the dose of prednisolone to the median of 40mg/day. In addition, immunosuppressants and biologic agents were commenced in 40 and 13 patients, respectively.

### Latent class analysis

Model selection criteria suggested that a multiple-group latent class model with two latent classes was preferable (Supplementary Table 2). All item response probabilities in the selected model are provided in Table 3 and additional information that characterizes these subgroups are summarized in Supplementary Table 3. Class 1 (*n*=155, 71.8%) was associated with a younger age of onset and typical symptoms that are included in the classification criteria, such as skin rash, arthralgia, and hepatosplenomegaly. On the other hand, Class 2 (*n*=61, 28.2%) was associated with higher serum ferritin levels, an older age of onset, and fewer typical AOSD symptoms. Frequencies of the major complications, MAS and DIC were not significantly different between Class 1 and Class 2 (*p*=0.50 for MAS and =0.10 for DIC). These data suggest that AOSD patients can be classified into two distinct subgroups, the typical AOSD group (Class 1) and the elderly-onset AOSD group (Class 2).

**Table 3:** Item response probabilities in latent class analysis

		Class 1	SE	Class 2	SE
		Estimate		Estimate	
White blood cell count (/μL)	≤6,000	0.10	0.03	0.05	0.03
	6000–12,000	0.42	0.05	0.29	0.09
	12,000<	0.48	0.05	0.67	0.10
Serum ferritin (ng/mL)	≤500	0.10	0.03	0.04	0.04
	500–5000	0.40	0.05	0.22	0.07
	5000<	0.50	0.05	0.74	0.08
Serum C-reactive protein (mg/dL)	≤0.3	0.01	0.01	0.00	0.00
	0.3–10	0.55	0.05	0.25	0.09
	10<	0.44	0.05	0.75	0.09
Age (years)	≤50	0.64	0.08	0.15	0.07
	50<	0.36	0.08	0.85	0.07
Splenomegaly	Presence	0.61	0.08	0.24	0.08
Hepatomegaly	Presence	0.40	0.07	0.13	0.06
Sore throat	Presence	0.68	0.05	0.53	0.08
Arthritis	Presence	0.82	0.04	0.75	0.07
Pericarditis	Presence	0.00	0.01	0.23	0.09
Pleuritis	Presence	0.01	0.01	0.37	0.12
Macrophage activating syndrome	Presence	0.20	0.04	0.27	0.07
Typical rash	Presence	0.64	0.05	0.37	0.1

*SE* standard error

### Survival and relapse

**Fig. 1:**
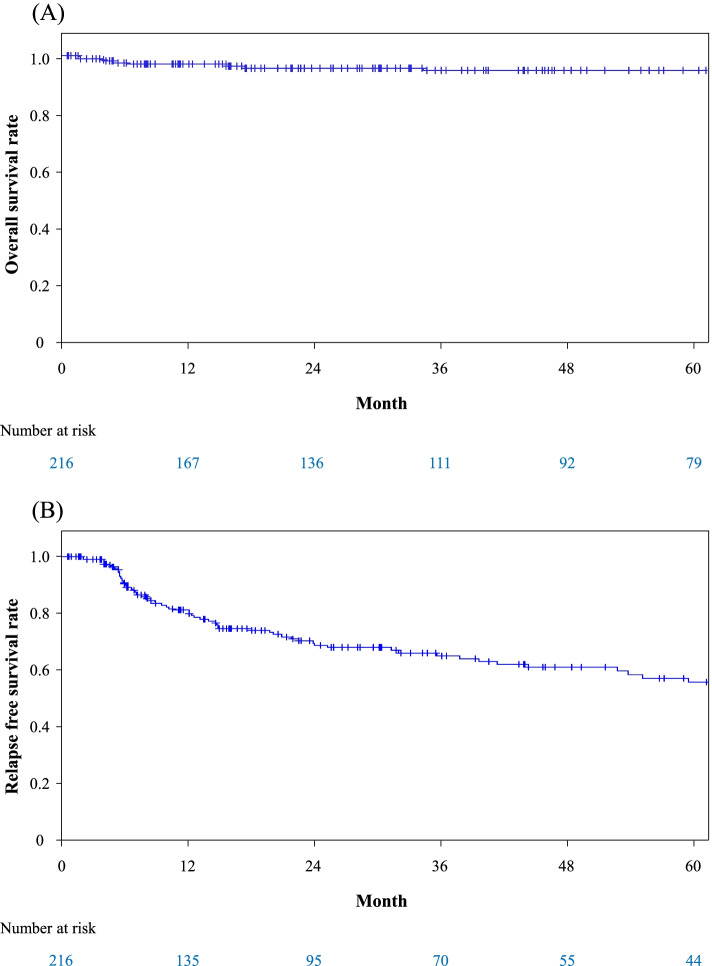
Overall and relapse-free survival rates of all patients. **A** Cumulative survival rate assessed by Kaplan-Meier survival curve is shown. **B** Cumulative relapse-free survival rate assessed by Kaplan-Meier survival curve is shown

During the follow-up period, 13 of 216 patients (6.0%) died (12 infections and one senility). Median age at death was 75.5 years. The overall survival rates at one and 5 years were 97.0% and 94.9%, respectively (Fig. 1A). We assessed Class 1/2 and all items in Tables 1 and 2 as potential risk factors for death. Univariate Cox regression analysis identified a higher neutrophil count, a lower hemoglobin level, and a higher age at disease onset as significant factors for death (*p*=0.02, 0.02, and *p*=0.01) (Table 4). In addition, Class 2 showed a tendency to a higher risk for death (*p*=0.05).

**Table 4:** Time to event analysis for death with Cox proportional hazard model

Parameter	Univariate analysis			
	HR	95%CI		*p* value
Neutrophil count, 10^3/μL	1.07	1.01	1.13	0.02
Hemoglobin, g/dL	0.69	0.50	0.95	0.02
Age ≤ 65 years old	Ref			
> 65 years old	45.40	5.80	355.61	0.01

*HR* hazard ratio, *95%CI* 95% confidence interval

Seventy-six of 216 patients (35.1%) experienced relapses, of which 33 (15.2%) had multiple relapses. Median time to the first relapse was 12.9 months. The relapse-free survival rates at one and 5 years were 81.6% and 57.3%, respectively (Fig. 1B). In univariate Cox regression analysis of relapse, only a higher age at disease onset was identified as a risk factor (*p*=0.01) (Table 5).

**Table 5:** Time to event analysis for relapse with Cox proportional hazard model

Parameter	Univariate analysis			
	HR	95%CI		*p* value
Age ≤ 65 years old	Ref			
> 65 years old	4.51	1.65	12.32	0.01

*HR* hazard ratio, *95%CI* 95% confidence interval

.

**Fig. 2:**
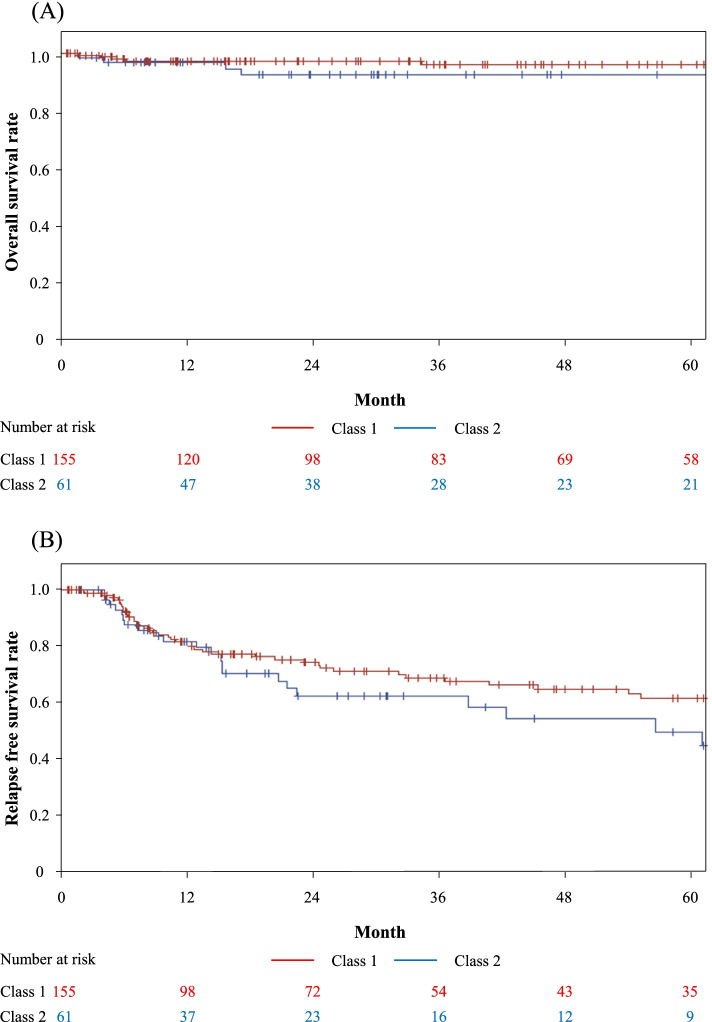
Overall and relapse-free survival rates according to Class 1 and Class 2. **A** Cumulative survival rates assessed by Kaplan-Meier survival curve are shown. Red line shows class 1 and blue line shows class 2. **B** Cumulative relapse-free survival rates assessed by Kaplan-Meier survival curve are shown. Red line shows class 1 and blue line shows class 2

Neither overall survival nor relapse-free survival rates were significantly different between the two classes (*p*=0.30 and *p*=0.19) (Fig. 2A, B) although patients in these two classes received similar treatments. Median initial doses of prednisolone were 42.5mg/day and 40mg/day in Class 1 and 2 (p>0.99). Sixty-nine of 155 (44.5%) in Class 1 and 27 of 61 (44.2%) in Class 2 received immunosuppressants (*p*>0.99), and 14 of 155 (9.0%) in Classes 1 and 8 of 61 (13.1%) in Class 2 received biologics (*p*=0.37).

## Discussion

AOSD is a rare systemic autoinflammatory disease and its etiology and pathology have not been fully understood yet. Diagnosis of AOSD is made on the basis of a set of non-specific clinical symptoms, and there is no specific laboratory marker for AOSD. Consequently, AOSD patients are heterogeneous populations and predicting their clinical courses in advance is difficult. We addressed these issues with two approaches, latent class analysis and time-to-event analysis using a large cohort of AOSD patients, both of which have not been reported in the literature. In this study, the latent class analysis revealed that AOSD patients were divided into two reasonable populations: the typical AOSD group (Class 1) and the elderly-onset AOSD group (Class 2). Treatment and outcomes were not significantly different between Class 1 and Class 2, indicating that treatment adjustment for each subgroup is not necessary. Thus, this subdivision may not directly help the current practice of AOSD patients. However, focusing on more specific subgroups may lead to attenuation of noise and facilitate finding new biomarkers when combining future studies, such as a genome-wide association study, a gene expression study and proteomics analysis.

The overall survival rate in this study was 94.9% at 5 years, which was comparable with previous reports [34, 35]. The time-to-event analysis with the Cox proportional hazard model expectedly identified age ≧65 years at disease onset as a risk factor for death. In contrast to the good overall survival rate, the long-term relapse-free survival rate was low (57.3% at 5 years). Although small studies in the past suggested that inadequate corticosteroid initiation doses, elevated white blood cell counts, high erythrocyte sedimentation rates, ferritin levels, and high systemic scores as risk factors for relapse of AOSD [6, 7, 36], only age ≧65 years at disease onset was identified as the risk factor for relapse in this study. The reasons for the relapse risk of older age are not known. Different intensity of treatments among ages might be the cause. Older patients in our cohort received similar initial treatments with younger patients; however, there was a possibility of more rapid reduction/withdrawal of prednisolone and immunosuppressants in elder patients considering treatment-related adverse events.

Regarding the classical classification of the disease courses of AOSD patients, 136 patients (62.9%) had a monocyclic pattern, 76 patients (35.1%) had a polycyclic pattern, and 4 patients (1.8%) had a chronic pattern in this study. The remarkable decrease in chronic pattern and increase in monocyclic pattern compared to previous reports [1] were observed in this study. Advances in the treatments of AOSD might be the reason for those changes in addition to differences in the local treatment strategies.

In terms of complications of AOSD, MAS was the most common in this study (22.3%), which was consistent with previous reports from Japan [37]. Wang et al. previously reported that the presence of MAS was the major cause of death in AOSD patients [38]; however, there were no deaths due to MAS in this study. In addition, there was no significant difference in death between AOSD patients with and without MAS in this study. These differences might be caused by the different treatment strategies between the cohorts. In our cohort, tocilizumab was frequently used in refractory cases, especially having MAS. Although there were reports that tocilizumab sometimes induced MAS in AOSD patients [39], among 31 AOSD patients who received tocilizumab in our study, none developed MAS after tocilizumab treatment.

In a previous study using a large US administrative claims database, acute respiratory distress syndrome was present in 12.3% of AOSD patients [40]. In the current study, on the other hand, interstitial pneumonia was observed in 5 patients (2.3%), which was the only pulmonary involvement. Another Japanese group also reported that the proportion of pulmonary involvement in Japanese AOSD patients was 2.5% [37], which was similar to our study. Pulmonary involvement is rare in Japanese patients with AOSD.

Recently, an Italian group published a study of similar concept to identify subgroups of AOSD patients. We used latent class analysis with a larger and Japanese cohort, while they approached using cluster analysis with an Italian population. They divided AOSD patients into four subgroups: cluster 1 with the highest ferritin levels, cluster 2 with the highest CRP levels, cluster 3 with the highest systemic scores, and cluster 4 with the lowest ferritin and CRP levels [41]. Although the reasons for the different results from ours were unknown, the different methods and patient populations might have led to the different results.

Patient data in this study consisted of only classical items; however, there are new modalities and biomarkers for evaluating the disease activity of AOSD. FDG-PET/CT was reported as a useful modality for evaluating the disease activity of AOSD [42], though not all facilities in this study have good access to FDG-PET/CT. Regarding biomarkers, elevated interleukin-1β, interleukin-6, and interleukin-18 were associated with a higher risk of MAS [12, 43]. Including those new items may improve the accuracy of the models by latent class analysis and Cox regression analysis in the future.

There were some limitations in this study. First, it was a retrospective study. There was no standardized treatment protocol, and treatments could influence outcomes as confounding factors. However, the multicenter design in this study might reduce treatment bias. Second, due to the small event number of deaths, we were unable to perform a multivariate analysis regarding the mortality risk. Third, our cohort lacked data about interleukin-1 inhibitors-use, though its efficacy for AOSD has already been demonstrated [44, 45]. Interleukin-1 inhibitors have not been approved for AOSD in Japan, while tocilizumab has been approved. Consequently, no patient in this study received interleukin-1 inhibitors.

## Conclusions

In this retrospective study, we assessed the large cohort of AOSD patients with the detailed dataset. Latent class analysis identified the two subgroups of AOSD patients: the typical group and the elderly-onset group. However, outcomes were not significantly different between the two groups in terms of overall survival and relapse-free survival. Although the survival of AOSD was generally good, one-third of the patients in our cohort experienced relapses. In this study, only age ≧65 years at disease onset was identified as the risk factor for relapse.

## Supplementary Information

**Additional file 1: Supplementary Table 1**. Patient characteristics according to presence/absence of macrophage activation syndrome. **Supplementary Table 2**. Selection of the number of classes in latent class analysis. **Supplementary Table 3**. Patient characteristics according to the latent classes.

## Data Availability

Anonymized patient level data can be made available on reasonable request and after approval by a review panel. Requests should be made to shfuruta@gmail.com.

## Abbreviations

AOSD: Adult-onset Still’s disease
sJIA: Systemic juvenile idiopathic arthritis
ASD: Adult Still’s disease
MAS: Macrophage activation syndrome
DIC: Disseminated intravascular coagulation
WBC: White blood cell count
Hb: Hemoglobin
ESR: Erythrocyte sedimentation rate
AST: Aspartate aminotransferase
ALT: Alanine aminotransferase
LDH: Lactate dehydrogenase
CRP: C-reactive protein
