# Supplementary material for: Latent class analysis of 216 patients with adult-onset Still’s disease
Source: Arthritis Res Ther. 2022 Jan 3;24:7. doi: 10.1186/s13075-021-02708-3 (PMC8722082; doi:10.1186/s13075-021-02708-3)
Supplement: Supplementary file 1 — Additional file 1: Supplementary Table 1. Patient characteristics according to presence/absence of macrophage activation syndrome. Supplementary Table 2. Selection of the number of classes in latent class analysis. Supplementary Table 3. Patient characteristics according to the latent classes. [file 13075_2021_2708_MOESM1_ESM.docx]

**Supplementary Table 1. Patient characteristics according to presence/absence of macrophage activation syndrome**

|  | Patients with MAS (n=48) | Patient without MAS (n=168) |
| --- | --- | --- |
| General characteristics |  |  |
| Age, median years [IQR] | 58.8 [38.0-71.2] | 66.8 [59.1-76.6] |
| Female, n (%) | 41 (85.4%) | 123 (73.2%) |
| Follow-up time, median months [IQR] | 33.4 [11.9-72.8] | 38.6 [14.9-82.6] |
| Proportion of patients with the specific symptom |  |  |
| Fever, % | 100% | 99.4% |
| Rash, % | 89.5% | 91.0% |
| Abnormal liver function tests, % | 81.2% | 80.9% |
| Arthralgia, % | 72.9% | 81.4% |
| Sore throat, % | 56.2% | 65.4% |
| Lymphadenopathy, % | 70.8% | 61.7% |
| Splenomegaly, % | 60.4% | 45.9% |
| Chest pain, % | 0.0% | 2.3% |
| Abdominal pain, % | 0.0% | 2.3% |
| Laboratory tests |  |  |
| White blood cell count, median /μL [IQR] | 12630 [8940-18350] | 12600 [9475-17800] |
| Neutrophil count, median /μL [IQR] | 10570 [7315-15866] | 10730 [7560-15762] |
| Hemoglobin, median g/dL [IQR] | 10.9 [10.0-11.5] | 11.1 [10.2-12.5] |
| Platelet, median /μL [IQR] | 141 [87-193] | 297 [197-386] |
| Erythrocyte sedimentation rate, median mm/hr [IQR] | 60 [31-95] | 77 [44-94] |
| Serum aspartate aminotransferase, median U/L [IQR] | 101 [63-220] | 61 [43-107] |
| Serum alanine aminotransferase, median U/L [IQR] | 58 [25-135] | 51 [30-100] |
| Serum lactate dehydrogenase, median U/L [IQR] | 762 [501-1183] | 478 [299-660] |
| Serum C-reactive protein, median mg/dL [IQR] | 10.8 [5.7-15.8] | 10.9 [5.7-17.2] |
| Serum ferritin, median ng/mL [IQR] | 18099 [9343-32145] | 4645 [1663-15150] |
| Complications |  |  |
| Disseminated intravascular coagulation, % | 35.4% | 7.1% |
| Renal dysfunction, % | 6.2% | 2.9% |
| Pleuritis, % | 18.7% | 10.7% |
| Pericarditis, % | 14.5% | 5.3% |
| Myocarditis, % | 0.0% | 0.0% |
| Interstitial pneumonia, % | 4.1% | 1.7% |
| *MAS; macrophage activation syndrome, IQR; interquartile range | | |

**Supplementary Table 2. Selection of the number of classes in latent class analysis**

| No. of Classes | BIC | cAIC |
| --- | --- | --- |
| 2 | 1119 | 1150 |
| 3 | 1145 | 1192 |
| 4 | 1153 | 1216 |
| 5 | 1189 | 1268 |
| 6 | 1263 | 1358 |
| 7 | 1332 | 1443 |
| *BIC; Baysian information criterion, cAIC; conditional Akaike information criterion | | |

**Supplementary Table 3. Patient characteristics according to the latent classes**

|  | Class 1 (n=156) | Class 2 (n=61) |
| --- | --- | --- |
| General characteristics |  |  |
| Age, median years [IQR] | 41.5 [30.2-58.1] | 66.8 [59.1-76.6] |
| Female, n (%) | 43 (70.4%) | 121 (78.0%) |
| Follow-up time, median months [IQR] | 18.8 [8.0-46.8] | 80.6 [40.0-135.8] |
| Proportion of patients with the specific symptom |  |  |
| Fever, % | 99.3% | 100% |
| Rash, % | 94.8% | 80.3% |
| Abnormal liver function tests, % | 82.0% | 77.0% |
| Arthralgia, % | 80.7% | 73.7% |
| Sore throat, % | 67.1% | 56.1% |
| Lymphadenopathy, % | 65.3% | 47.5% |
| Splenomegaly, % | 58.3% | 18.0% |
| Chest pain, % | 0.0% | 6.5% |
| Abdominal pain, % | 0.6% | 4.9% |
| Laboratory tests |  |  |
| White blood cell count, median /μL [IQR] | 11700 [9100-16700] | 14804 [10800-20000] |
| Neutrophil count, median /μL [IQR] | 9648 [7232-14098] | 13660 [9288-18612] |
| Hemoglobin, median g/dL [IQR] | 11.2 [10.2-12.5] | 10.6 [9.7-11.6] |
| Platelet, median /μL [IQR] | 271 [167-358] | 239 [159-384] |
| Erythrocyte sedimentation rate, median mm/hr [IQR] | 73 [38-95] | 79 [56-94] |
| Serum aspartate aminotransferase, median U/L [IQR] | 61 [44-121] | 79 [54-141] |
| Serum alanine aminotransferase, median U/L [IQR] | 52 [30-105] | 52 [30-100] |
| Serum lactate dehydrogenase, median U/L [IQR] | 504 [306-743] | 568 [332-783] |
| Serum C-reactive protein, median mg/dL [IQR] | 8.9 [4.6-14.3] | 14.7 [11.0-19.5] |
| Serum ferritin, median ng/mL [IQR] | 4443 [1606-16302] | 14810 [5846-30000] |
| Complications |  |  |
| Macrophage activation syndrome, % | 20.6% | 26.2% |
| Disseminated intravascular coagulation, % | 10.9% | 19.6% |
| Renal dysfunction, % | 3.2% | 4.9% |
| Pleuritis, % | 1.2% | 4.1% |
| Pericarditis, % | 0.0% | 2.6% |
| Myocarditis, % | 0.0% | 0.0% |
| Interstitial pneumonia, % | 1.9% | 3.2% |
| *IQR; interquartile range |  |  |
